# Mourning and orienting to the future in a liminal occasion: (Re)defining British national identity after Queen Elizabeth II's death

**DOI:** 10.1111/bjso.12807

**Published:** 2024-10-08

**Authors:** Sandra Obradović, Nuria Martinez, Nandita Dhanda, Sidney Bode, Evangelos Ntontis, Mhairi Bowe, Stephen Reicher, Klara Jurstakova, Jazmin Kane, Sara Vestergren

**Affiliations:** ^1^ School of Psychology & Counselling The Open University Milton Keynes UK; ^2^ School of Psychology and Life Sciences Canterbury Christ Church University Canterbury UK; ^3^ Geneva School of Economics and Management University of Geneva Geneva Switzerland; ^4^ School of Psychology and Neuroscience University of St Andrews Andrews UK; ^5^ Department of Psychology Heriot‐Watt University Edinburgh UK; ^6^ School of Psychology Keele University Keele UK

**Keywords:** ethnography, liminality, national identity, Queen Elizabeth, rituals, sense‐making

## Abstract

In this paper, we conceptualize the days of mourning that followed the passing of Queen Elizabeth II. as constituting a liminal occasion, a moment of in‐betweenness through which we can explore sense‐making in times of transition. How do people navigate through liminal occasions, and are they always transformative? Through a rapid response ethnography (*N*_interviews_ = 64, *N*_participants_ = 122), we were able to capture the raw moments within which a collective comes together, as part of a national ritual, to transition from ‘here’ to ‘there’. In our data, liminality prompted participants to strategically define British national identity and its future by positioning the Queen as representative of Britishness, her loss as a national identity loss. No longer taken for granted, participants reasserted the value of the monarchy as an apolitical and unifying feature in an otherwise divided society, characterizing the continuity of the institution as an essential part of British identity and society. The analysis illustrates how liminality offers a useful conceptual tool for addressing how temporality and change are negotiated in relation to a shared identity, and how navigating transitional moments brings with it political implications for the future.

## INTRODUCTION

On 8th September 2022, Queen Elizabeth II., the UK's longest reigning monarch, died after more than 70 years on the throne. Her death was marked by a ten‐day period of mourning. During this period, over 250,000 people queued to file past her coffin in Westminster Hall, a million attended the funeral procession, some 29 million people in the United Kingdom watched on television, and 4 billion watched worldwide. This was a mass event (or else, a series of mass events) of truly global proportions, experienced by crowds of mourners. Over recent decades, there has been a resurgence of psychological interest in the study of crowds – how they reflect and reconfigure social identities. However, this has generally focused on contentious crowd events: demonstrations, riots, sporting disturbances and so on (Drury & Reicher, 2021; Reicher, 2001). Psychologists have paid far less attention to consensus events, such as ceremonials and commemorations that affirm and celebrate the status quo. By contrast, historians and anthropologists have done more to study the societal relevance of these (e.g. Galvin, 1998; Gillis, 1996; Ozouf, 1991) considering these events as also serving important means of reflecting and reconfiguring identities. Whether contentious or celebratory, crowd events always project into the present a desire for a particular imagined future.

This paper forms part of a larger project concerned with exploring who participated in the events following the death of the Queen, how they experienced their participation and the impact it had on their understanding of themselves and of their Britishness. In this paper, we conceptualize the days of mourning that followed the passing of Queen Elizabeth II. as constituting a liminal occasion (Stenner, 2017), a moment of in‐betweenness through which we can explore sense‐making around social identities in times of transition and examine what kind of futures are imagined, or become possible (de Saint‐Laurent et al., 2018). Drawing on Stenner's theory of liminality (Stenner, 2017), we examine how mourners navigated this liminal experience in a socially guided and culturally mediated way: how did participation serve to (re)affirm and (re)define their understanding of the monarchy, of its relevance to British national identity and the country's future.

## LIMINALITY AND (COLLECTIVE) RITES OF PASSAGE

Liminality refers to the ‘in‐between’ stage within a transitional moment: a time and space where one is ‘no longer’ but also ‘not yet’ (Larson, 2014). The concept offers a useful avenue for capturing the circularity of temporality and the future‐oriented nature of group actions. It explicitly focuses on moments of uncertainty, ambivalence and tension. In those possibility‐rich spaces, imagining the future becomes a powerful way to navigate an unstable and transitional present, oftentimes, by bringing back a nostalgic and long‐gone past.

The concept of the ‘liminal’ originated in the work of anthropologist Arnold Van Gennep (1960). Van Gennep's book, *The Rites of Passage*, introduces the word ‘liminal’ to conceptualize transition rites. In reviewing anthropological and historical data across languages and contexts, van Gennep identified a ‘pattern of rites of passage’; transitions were navigated across three kinds of ritual ceremonies: rites of separation, rites of transition and rites of incorporation (Stenner, 2021). Victor Turner (1967) further developed and extended the concept of ‘liminality’ during the 1960s. Turner (1969) emphasized how liminal occasions involved the temporary and ritual suspension of social structure, where differences of status, identity and rank are for a short, but intense time, uprooted. He referred to these liminal phases as defined by ‘anti‐structure’. In unsettling what is taken‐for‐granted, liminal occasions destabilize identities and require collectives to engage in active labour to transition from ‘here’ to ‘there’, a process that relies on group meaning‐making (Zulato et al., 2023). Conceptualizing the context after the Queen's death as a liminal occasion enables us to examine how individuals make sense of the implications of this loss for their national identity and culture, but also how they *use* the ambiguity within the liminal occasion to propose their own articulations of what the loss means, what the past was and what the future should hold. This highlights how liminality becomes a useful concept to locate within the broader social identity framework. By examining meaning‐making within liminal occasions, we can identify and analyse the political and national identity projects that ingroup members seek to mobilize for the future. After all, not only are social identities not fixed in time and space, but also, when subjects speak, they do not simply reflect stable internal cognitive elements that point to underlying identities – instead, multiple versions of identities are possible and can be articulated by reflective social actors with particular agendas towards the future (Reicher & Hopkins, 2001). Liminal occasions, where the taken‐for‐granted becomes uprooted, provide key moments through which the active process of reproducing and reconstructing characteristics of national identity occurs, and becomes consequential, for the future.

Liminal experiences can occur in response to both spontaneous and devised liminal occasions (Stenner, 2017). The former refers to events which happen *to us* unexpectedly and unpredictably (such as the death of a loved one), while the latter refers to events *devised by us* usually in defined time/space which are mediated by cultural resources to mark a transition (i.e. winter solstice as a form of seasonal transition). Of particular interest to social psychologists should be the relationship between spontaneous and devised liminal experiences. For example, arguably, the death of Queen Elizabeth II. can be considered a spontaneous liminal experience, which ruptured the everyday lives and worlds of people in the United Kingdom (and beyond). While the formal transition from Queen to King (King Charles III) was immediate, the public engaged in a series of ceremonies and rituals between September 2022 (Lying‐in‐State; National days of mourning) and July 2023 (Coronation) which marked a transitional period between the ‘no longer’ reign of Elizabeth II and the ‘not yet’ reign of Charles III (Larson, 2014). During this time, questions around what Britishness was and would be in the future were core to the public debate (see Freedland, 2022) and created a ‘liminal occasion’ through which who ‘we’ are was being navigated.

This liminal period included multiple ceremonies and rituals which took a highly structured form. Much of the British public actively participated in commemorating Queen Elizabeth II's death in schools, workplaces, communities and in their homes. The significance of the event was evidenced by thousands and millions of mourners across the United Kingdom who participated in the national days of mourning in various ways. There are parallels that can be drawn between this modern phenomenon and a pre‐modern one, pilgrimage. Victor Turner considered pilgrimage as liminality exemplified, an event full of possibilities for the traveller by becoming a ‘world within a world’ (Stenner, 2021). Through pilgrimage groups can ‘experience personal and social transformations by literally and figuratively moving beyond the structures, social statuses, norms, routines, and rhythms that regulate daily life’. (Beckstead, 2021, p. 102). In discussing pilgrimage, Scriven (2020) emphasizes how the path travelled shapes the relationship between pilgrims by offering a ‘connective tissue’ permeating movements and reproducing a sense of shared identity in the process (p. 269). Not only this, but pilgrimages can also serve to change and strengthen core identities that structure everyday life (in this case, Hindu identity – see Hopkins et al., 2019) and reduce outgroup prejudice by nature of contact (Alnabulsi et al., 2020).

Likening the mourning events as a transitional moment, navigated through a form of modern‐day pilgrimage set on a national stage, we are presented with a theoretical lens for studying affirmations of, and changes in, British national identity. Both elements – continuity and change – are important, and it is important not to emphasize the one to the exclusion of the other. Indeed, a key element of liminal occasions and of the ritualized elements that constitute them is precisely a tension between possibilities for change and attempts to maintain a sense of stability. As Stenner (2021, p. 25) argues, according to Van Gennep (1960) ‘the “function” of rites of passage is to “reduce the harmful effects” of disturbances to the life of society caused by actual changes of conditions amongst its members’. Through state‐organized rituals and ceremonies, spontaneous liminal experiences can become culturally organized through shared social practice, becoming instead devised liminal experiences aimed to ‘bring order into the liminality of spontaneous events’ (Wagoner & Zittoun, 2021 p. ix). As such, the ceremonies and rituals that featured in the national days of mourning functioned to turn spontaneous liminal experiences into devised ones, controlling how this transitional movement was navigated. Hence, although the institutionalized ‘suspension’ in time destabilized the routine day‐to‐day life of citizens, it also, by nature of being ‘institutional’, scaffolded the transition from ‘here’ to ‘there’ in a way that aimed to reaffirm, rather than contest, the relevance of the monarchy for the future.

Beckstead (2021, p.90) sums up the nature of the continuity/change tension, ‘Liminal experiences, therefore, may alter the social order or they may serve to further instantiate the social order in which they temporarily upend and bind the subject more tightly to this order’. As we will show, it is through the crowd participating in the ceremony that a collective transitions through a liminal experience together, where destabilized identities are resolidified and order ultimately reaffirmed rather than challenged.

## LIMINAL OCCASIONS AND PERCEIVED COLLECTIVE CONTINUITY

For social psychology, liminality becomes a useful concept for examining the paradox of sameness within change; the human ability to experience a seemingly natural and continuous movement of a collective across time (i.e. the perceived collective continuity of a group; Sani et al., 2007) while simultaneously acknowledging the inevitability of change. As Stenner (2017, 2021) argues, liminality is ‘a world within and between worlds’ that entails both transitioning between worlds and the transformation of worlds. Conceptually, then, liminality offers a theoretical account of transitions which enables social psychologists to move beyond atemporal and static studies of social phenomena to capture the experience and possibility inherent in moving between present and future states.

Previous research has shown that when groups experience an event as threatening to the nature or continuity of group identity, members often look to the past for solutions (Smeekes & Verkuyten, 2015; Wohl et al., 2022). As Stenner (2021) explains, a liminal occasion prompts a period of uncertainty ‘which can disrupt expectations for the future, re‐configure memories of the past, and thereby transform the very seat of reality: the present’ (p.5). This uncertainty means liminal occasions become moments where nostalgia thrives (Davis, 1979). Arguably, given the central role of the monarchy for lay understandings of British identity (i.e. Olechnowicz, 2007; Stevenson & Abell, 2011; Tranter & Donoghue, 2021) the loss of a monarch means not only the loss of a cherished individual and of someone who represented the nation to the world, but also a potential loss of the collective itself. This multiple sense of loss can trigger reflections regarding the national past. In these moments, collective nostalgia serves a future‐orienting and stabilizing function (Wohl et al., 2022). By reflecting on a stable past, group members can identify the elements of group identity that need protecting or repairing and which should therefore be advanced in the future. Yet, this future‐oriented emphasis is itself subtle, implicitly embedded in how the past is drawn into the present, namely what we choose to selectively remember about the past and be nostalgic for (Wohl et al., 2022). Billig (2002, p. 213) makes this point when discussing the ways in which the past is brought into the present, emphasizing that in this process there is a subtle, but important omission; ‘the future slips from the argumentative agenda. It becomes a blank category, whose nature is to be extrapolated from the gap between the past and the present’. The cyclical relationship between the past, present and future is also evidenced in the literature on reactionary politics (i.e. Capelos & Katsanidou, 2018; Sullivan, 2021) which further attests to the intricate relationship between how imagined futures are often affectively ‘carved out’ with reference to distant pasts.

## THE QUEEN'S DEATH IN CONTEXT

Through the present study, our aim was to explore how those attending national mourning events navigated the rupture created by the death of Queen Elizabeth II. by examining how they made sense of her passing and its implication for British identity and the perceived future of the United Kingdom. We have argued that her death can be seen as creating a liminal occasion where the ceremonies and rituals held during the 10 days of mourning liken a modern‐day pilgrimage, a shared cultural experience that is socially organized to shape how the collective transitions from ‘here’ to ‘there’.

Queen Elizabeth II. reigned as the Queen of England for 70 years, making her the longest‐reigning monarch in British history, the only monarch recognized and experienced by most Britain's citizens. For them, her death was an unprecedented occasion, not the death of *a* monarch but the death of *the* monarch. And for many, who identified as British and identified Britishness through the monarch, it placed their own selves and their own future in question – creating a liminal occasion. In this in‐between period, how did people make sense of who they were, how did they anchor themselves in the past while looking to the future? Moreover, how did they do this collectively through the ritualized crowd events of the mourning period? These are the questions we seek to address in this paper by analysing voices from these crowds.

## METHOD

### Study design

This study used an ethnographic approach to data collection to allow for an exploration of the national phenomenon of collective mourning following the death of Queen Elizabeth II in September 2022. Following the announcement of Her Majesty's death, a team of researchers from across nine universities assembled to shape and plan a study of participants' experiences, motives and accounts of taking part in the planned 10 days of national mourning. The ethnographic approach chosen allowed for the collection of a range of data sources from within the collective events, including interviews, photographs, videos, field notes and voice memos (see, e.g. Hammersley & Atkinson, 2019; Vestergren & Drury, 2020). Given the aims of this paper, we draw only on the data gathered from short, on‐site interviews with participants in the queue to explore their accounts of sense‐making during this liminal period and, in particular, what these accounts were ‘doing’ in constructing the national character and the future of the nation.

### Participants and procedure

Utilizing a rapid response ethnography (for additional information of how this was ethically accomplished, see Hoerst et al., 2022), data collection commenced in Edinburgh on 12th September 2022, when crowds formed on the Royal Mile and on the streets approaching St Giles Cathedral and progressed in London during the queueing on the approach to Westminster Hall, before culminating within the gathering of crowds on the day of the Queen's funeral at public screenings and locations across London.

An opportunistic sampling approach was utilized which involved researchers (typically in pairs) approaching members of the public to take part in the research. Following a brief introduction to the study, where researchers identified themselves and their interest in public participation in the collective mourning events, crowd members were asked if they would like to answer a few questions about their experiences and if they consented to being recorded. Where consent was granted, researchers informed participants of their right to halt the interview at any time, of the nature of data storage, use, and security, and of steps taken to protect anonymity and confidentiality (see Appendix S1 for interview guide). Researchers then recorded short interviews using mobile recording devices, which ranged from brief ‘vox pop’ recordings to longer interviews (typically during uneventful periods, e.g. while waiting in the queues). Interviews lasted between 1 minute and 24 min, with an average length of 6 min per interview (see Appendix S1 for more information).

Each conversation was guided by a series of pre‐decided questions agreed upon by the research team. These covered topics such as participants' reasons for attendance and further details (e.g. commute information, whether they were attending with others or alone), relationships with other crowd members, perceptions of the crowd and any potential impact of participation. Depending on who was approached, data were collected from individuals, pairs or small groups of participants. Throughout data collection, researchers were cognisant of the potentially upsetting nature of the events and monitored participants for any signs of distress. At the culmination of all discussions, participants were asked if they had any questions, and were then thanked for taking part. This approach resulted in data collected from 122 participants in the context of 64 short interviews.

### Analytic procedure

Data were collected by some members of the team but fully transcribed and read by all researchers. Preliminary reflections on the data were recorded and shared during team meetings with reference to initial codes. This process revealed features of the data involving the historical framing of the Queen and temporal links between past, present and future. Following this discussion, we developed a clear set of aims for the analysis in relation to this topic. In‐depth coding of the whole data corpus was then conducted by the first and second authors. Coding focused on capturing sense‐making through the participants' responses, focusing on how they positioned the Queen's death in relation to the collective (rather than only their own sense of loss or grief). We focused on analysing instances where temporal references were used, where the past, present and future were discussed and how participants linked their strategic construction of national identity with the Queen (and her passing). From this, two key themes aimed at addressing the research aims were developed and refined by the first two authors. These were subsequently discussed and reviewed by the core team (the first six authors and the last author). Ongoing collaborative discussions throughout the analytical process enabled us to ensure the quality, robustness and trustworthiness of the findings (Levitt et al., 2021).

## POSITIONALITY AND REFLEXIVE OPENNESS

For reflexive openness (Braun & Clarke, 2024), we want to acknowledge that our research team included both British and non‐British researchers, something which we see as beneficial for data collection and analysis. While non‐British researchers were able to elicit in‐depth responses among participants (giving details that they would not provide to British researchers who they assume would have shared knowledge), British researchers, on the other hand, were able to provide the cultural insights to the data. This mix, alongside the inclusion of researchers at the analysis stage who had not collected any of the data themselves, brought in multiple critical perspectives allowing for both informed and challenging interpretations of the data.

## ANALYSIS

The analysis below is divided into two interconnected sections. First, we examine whether the death of Queen Elizabeth II created and was perceived as a liminal occasion and what kind of sense‐making was triggered as part of a liminal experience. Secondly, we identify the rhetorical and ideological functions of liminal occasions in (re)defining British national identity against the backdrop of an imagined threatening future. Here we argue that liminal occasions can be constructed as both narrow and wide, opening up more, or less, potential for transitions to be transformative, or reaffirming, of social identities.

### The Queen's death as a period of transition and future possibilities

The experience of liminality was evident in the responses of those in the queues in Edinburgh and London. This mainly occurred through the use of temporal language that emphasized the Queen as a positive and constant fixture of British society and life (e.g. ‘she's been part of our lives forever’), her constant and predictable presence in important collective rituals (i.e. Christmas and her Christmas speech, Oaths of allegiance), in family traditions bringing generations together (making royal ‘scrapbooks’, watching royal events together on television) and in everyday symbols (e.g. money, stamps, songs and emblems). Consequently, her death triggered sense‐making around the continuity and character of the national group moving forward:

Extract 1: ‘We've never had, we've only known the Queen, so all our lives my dad has only known the Queen. And‐and now you think about changing the money, changing everything, I'm like ‘God Save the King’. [I: yeah; I2: yeah] everyone wants stability. It's like everything's changed now, like someone pulling the rug from under you. (…) It's an end, a new era’. (S2, London, I‐53)

The above extract illustrates what Stenner (2017) refers to as an ‘uh oh!’ moment, a response to a spontaneous liminal experience that triggers a realization that much of what was previously taken for granted (the presence of the Queen in multiple aspects of everyday life through rituals and symbols) now requires attention and requires us to make sense of our world in the absence of the familiar. Even though the possibility of the Queen dying was a possible future that would, inevitably, come one day, there is still an element of shock and surprise evident by the simile of having the ‘rug’ pulled from under them. A similar sense of shock was present in other accounts (e.g. ‘I always knew that I was coming, that was never a question. I think it was a bit of a… shock at how‐how quick it was’., S1, London, I‐31).

Talking about the currency and the national anthem, in this passage, we can see how this speaker indicates that ‘everything’ will be changed and transformed in this journey between the different eras, ‘signalling a suspension of time/space that is indicative of liminal experiences as a “world within and between worlds”’ (Stenner, 2021). Indeed, in our data, many participants described how the Queen's death had brought about an end to an era. As later quotes will evidence, what is at ‘stake’ within this liminal occasion is better understood by situating her death within a broader socio‐political context, where the sense of ‘end’ includes the ultimate loss of a point of stability across a period of many changes, fractures, and tensions.

Interestingly, however, for many participants, the liminal occasion created by the Queen's death was not only a moment of loss, but a time when the national values, beliefs, attitudes and traditions embodied by the Queen could resurface and hopefully become re‐asserted in society. This highlights how the ambivalence experienced as part of liminality takes on an affective dimension as well; while mourners were sad and shocked, they were also hopeful, proud and, as in the Extract below, emboldened:

Extract 2: Speaker 1: Yeah, I think the fact that so many thousands, millions of people are so involved in this is, is because she represented something of who we were. And as I was sort of saying at the beginning, that sort of slight feeling that we might be losing track of who we are. And so, I think if the Queen's death has done something positive and good, it could be that it's brought us back to an understanding of who we are or who we're meant to be as a nation. And…

Interviewer: Can I ask you to elaborate on that a little bit more. Who are we?

Speaker 2: We apologise a lot for who we are nowadays, and perhaps we should do a little less apologising about our past.

Speaker 1: Be a bit more proud.

(London, Interview 37)

We see mourners articulate a clear link between national identity and the Queen as she ‘represented something of who we were’ (note the past sense). Her death, in turn, forces the collective into a liminal experience, an ambiguous moment triggering sense‐making ‘of who we are meant to be as a nation’. The liminal occasion offers an opportunity to assert a particular national identity project for the future, evident by the reference to the incompleteness of identity (‘meant to be’). The liminal occasion opens up what is perceived by participants as a legitimate space to express national pride and assert a particular version of national identity that in other spaces could be perceived as improper and hostile. The Queen's death, while tragic, brings with it a moment of hope, where the solution to identity rupture is retrospection; where ‘who we were’ has the potential to be ‘brought […] back’. The temporal words used to indicate that the future is one of returning to a better version of British identity as located in a selected past. Put differently, in the liminal occasion created by the Queen's passing, the necessity of cultural rediscovery and preservation (Lowenthal, 1985) of the ‘essence’ of the national group was stressed against the backdrop of an imagined threatening future.

This illustrates the constructive nature of national identity projects, with a selective reading of *what* past we bring into the future. The assertion of expressing more pride and less apologizing situates sense‐making of the Queen's death within a broader socio‐political landscape; one marked by tensions and a fractured public emerging from the Brexit referendum, and more recently the Black Lives Matter protests, the toppling of the Colston statue in Bristol and the recent report on racism, misogyny, and homophobia in the police. Instead, this moment of loss and mourning warrants expressions of British nationalism to be justified as pride by becoming rooted in (a selective reading of) history that identifies an alternative, and more positive national identity project as existing ‘before’, and as such, being possible again. How the Queen, and a temporally enduring monarchy, embody this alternative, was echoed in other interviews:

Extract 3: I mean, she, she's unique. She spanned two eras…really. The British have 1000‐year history of monarchs, right. And so, she seems her character was forged in the second year of World War. And when the Brits were standing so strong and doing the right thing, and she picked up that thread, and of the monarchy and that string and doing the right thing. And since then, she has always done the good and like, [I: okay, yeah]. Which I think is marvellous. (London, Interview 26)

In this extract, the participant draws on a historical narrative to construct a temporally enduring (1000 year) relationship between the national group (‘The British’) and the monarchy. This narrative is used for two purposes. On the one hand, it serves to define the monarchy as a constant and important feature of the British nation (Nairn, 1994), thus potentially undermining any counterarguments that the monarchy is separate from, or not representative of, the national group. On the other hand, this account also serves to represent the Queen as a gatekeeper between the present and the past, as the hinge between ‘two eras’. The Queen, whose ‘character was forged in the second year of World War’, is seen as ‘picking up’ on the national identity that was there (‘the Brits were standing so strong and doing the right thing’) and embodying it through her leadership. This, in turn, both positions the Queen as a prototype of a positive British character situated in the past, and as an exceptional (‘unique’) persona in the present, by means of particularization (Billig, 1985). The uniqueness of her character is defined by her ability to embody a seminal historical period (‘World War Two’) that the participant associates with displays of national strength and righteousness. These values are implied to be under threat in present times through the use of past tense (‘when the Brits were’) and the implications of the loss of a leader who maintained these values over time (‘she has always done the good’). Consequently, the liminal occasion becomes an opportunity to engage in a collective ritual where the public ‘pick up the thread’ of core values carried by the Queen ‘before’, and bring them into the ‘after’, a British future without the Queen.

This sense of public responsibility to ensure temporal connectedness in times of perceived loss and change is clearly displayed in the following extract:

Extract 4: She was part of the greatest generation. And it's sad to see her go, because she symbolised a lot of grace and elegance and dignity of that time, which I think is sometimes you lose that. But I feel in this, in this time right now, all of those values for what she stood for are now coming to the forefront, and people are recognising them, and the attributes that made her a great queen. And I think those are just what unites people coming to see her, people long for that grace, that dignity, elegance, the ability to unify people, not be partisan. (London, Interview 43).

In this quote, the participant is invoking a nostalgic and glorified image of the national past (‘she was part of the greatest generation’) to articulate the Queen as an embodiment of national values of better past times (‘grace and elegance and dignity of that time’) that are defined as currently being under threat (‘sometimes you lose that’). What is implied through the response is the link between longing and belonging; the values that the Queen stood for, rooted in the past, are also the same ones that in the present ‘unite people coming to see her’. It combines the unifying function of national nostalgia (Davis, 1979; Smeekes & Verkuyten, 2015) with the uses of history during times of transition (Hobsbawm, 1992) and utopian vision to characterize the crowd and assert its unity. People have come, not to bury the Queen, nor even to praise her, but to appropriate her greatness for their future.

### Legitimizing the monarchy in transition: A unifying and ‘apolitical’ British symbol

Recurrent across our data set was also a definition of the Queen and, for some, the monarchy, as emblems of unity by means of their perceived political neutrality and, hence, ability to represent everyone within the country irrespective of ideology. As we will see, rhetorically, this allowed some participants to legitimize the monarch as an undisputed and unproblematic symbol of national unity and, thus, for viability of the nation:

Extract 5: She was a very stabilising figure, she was not polarising, in the sense that she could not express a view. But I think by doing so, she made people care for her and love her because she rose above politics. (London, Interview 43).

Here, the Queen is being defined as an impartial, apolitical leader. The Queen's seeming inability to express political sentiments is also interpreted as the reason why she managed to enjoy a widespread sense of admiration and support within the country (‘she rose above politics’). In the extract below, her perceived apoliticality is equated to her unifying character:

Extract 6: I think I've learned quite a lot about the monarchy in the last week. I think it, I mean…I think I've learned that its significance and importance more than I even recognised before then. It's a key for me, it represents a total piece of glue in our society that's above politics. So, the people here will have very different political views and some of them quite polarised. But the monarch represents something that we all are, have, have an affinity for. So, it's above the politics and therefore it's a sense of, of communal that like nothing else in this country. It's the single thing which brings people together. (London, Interview 64).

Within this quote, the participant is reflecting on a renewed insight into the national importance of the royal family after the Queen's passing (‘I've learned the significance and importance more than even recognised before’). As liminal occasions become ambiguous states between ‘before’ and ‘after’, they also necessitate a collective sense‐making process to identify what will characterize the collective when it moves out of a liminal state. In the quote above, we see that for this participant, the days of mourning have reaffirmed, rather than challenged, the role of the monarchy in the national character as it is the monarchy itself, rather than the Queen, that is crucial to the collective. By positioning the Queen as an extension of the monarchy, this limits the transformative potential of the liminal occasion by narrowing how much of a ‘rupture’ it causes; the loss of the Queen is sad, but it stands against the legacy of a long‐lasting, and continuing, monarchy.

The extract further echoes Scriven's (2020, p. 269) points about how the path of pilgrimage creates a ‘connective tissue permeating our movements’. This is evidenced by the reference to the physical presence of a collective ‘the people here’ which follows from a reference to the monarchy representing ‘a total piece of glue in our society’. The physical movement through a shared space literally facilitates the liminal experience as a form of passage between one world and another (Stenner, 2021), yet it is not transformative in its challenging of the authority of the monarchy, but rather the transformative moment comes from realizing and reaffirming a potentially previously taken‐for‐granted, or less visible, significance of the monarchy for British identity. This echoes Abell and Stevenson's (2011) observation that vernacular representations of the British monarchy justify its maintenance by reference to its defined role as a ‘social glue holding together diverse populations’ (p. 488) within the country. In this extract, its unifying potential goes further in suggesting that the monarchy creates a feeling of superordinate belonging that cuts across political cleavages and polarization (it is ‘*above politics*’, ‘it represents something we all have an affinity for’), too. Contrasting the unifying character of the monarchy to the divisive nature of politics, the extract ends denoting, once again, the unique role that the monarchy has in creating national unity, as the participant asserts that the royal family enjoys generalized support throughout the nation (‘it's the single thing that brings people together’). Consequently, any threats to the role of the monarchy for the future of Britain, is a threat to the ‘single thing’ that, in a socio‐political context of fractures and tensions, is holding it all together.

Extract 7: Interviewer: What do you think? Do you think that this relates to society as a whole this event, like people being here, how do you think that reflects on society?

Speaker: Oh, 100%, I think it's about…it's about the nation coming together to pay their respects to someone who's really important, historically important to us. And yeah, part of me feels as if I hope, you know, I've got I've got hope that, you know, the bitterness of politics maybe over the last couple of years will sort of dissipate a little bit, and there'll be more of a sense of unity and coming together. And actually, you know, bringing people together a little bit more, maybe a little bit more consensus in politics, which is something I would like to see rather than the sort of…sharp division both in Scotland and also in the Westminster that I think we've all experienced over the last two, three years. (Edinburgh, Interview 18)

Liminal occasions create opportunities to renegotiate and redirect the course of the future. In the exchange above, a queuer in Edinburgh expresses their hopes that the future will be one of more unity and less political division, situating the Queen's death as a potential ‘end’ to a time‐period of conflict and division. This, in turn, is achieved when ‘the nation’ comes together, something it supposedly had done as part of the rituals of mourning. Through references to shared experiences as ‘bringing people together’ and potentially creating ‘a bit more consensus’, the participant presents the Queen as uniting the public above and beyond politics. Yet, participants did attribute political intentions to the Queen, especially around the issue of the British union, among those attending the Edinburgh memorial. For attendees of the mourning events, the Queen was not only seen to be the representative of Scotland as part of Britain, but her death was presented as portraying a particular political message. This was implicitly done in a variety of ways, as seen in the quote below:

Extract 8: I'm a massive royalist. I'm also a massive unionist so I think there should be a union together and I think it's really symbolic that the Queen has chosen, or this has happened in Scotland, and I think it's her last act of union to me as this has happened in Scotland and Scotland has set the stage and again, seen on TV. I mean, it's just the most beautiful country in the world. [I: (….)]. With the Scottish flag over the coffin and the bagpipes and all that [inaudible] but yeah, she's the Queen of Scots; she is the Scottish Queen. 100 percent. (Edinburgh, Interview 5).

This extract ascribes unionist and unifying attributes to the Queen in several ways. Firstly, the quote defines the Queen as a symbol of the Union by framing her passing in Scotland as an intentional act (‘the Queen has chosen’), matching the political views of the interviewee. In this account, the participant also attributes political attitudes to the Queen by hinting at a sequence of unionist acts she has performed during her reign, with her ‘*choosing*’ Scotland as being the ‘last act of union’ out of a series of non‐mentioned deeds. Secondly, beyond being depicted as a symbol of unionism, the final lines of the quote also construct the Queen as an unambiguous symbol, and leader, of a distinctive Scotland (‘she's the Queen of Scots’; ‘the Scottish Queen’), using the expression ‘100 percent’. The national singularity of Scotland is emphasized when the participant talks about the ritualistic aspects of the funeral (‘*flag*’, ‘*bagpipes*’ and ‘*all that*’) which, in turn, give visibility to such distinctiveness within the broader polity. Similar arguments are invoked in the next extract:

Extract 9: Speaker 1: I think the fact she was in Scotland as well, I mean …with independence. [I‐ Yeah.]

Speaker 2: And it's very good, very good in a bad way that she's died in Scotland. It's given Scotland now, it's opened up to the world, how beautiful Scotland is and how beautiful Edinburgh is, I mean, some of these aerial views you saw of the city yesterday and all the way down the road. It's amazing.

Speaker 1: And I also think we'll find, hopefully, bring Scotland back together again with the United Kingdom and maybe turn a lot of people. Get away from all this referendum nonsense

Speaker 2: I think that's the Queen's last gift to the country. (Edinburgh, Interview 10)

Beyond attributing unionist attitudes to the Queen, this extract also highlights how, in these participants' discourses, the Queen's death in Scotland was also associated with a sense of hope for the political future of the Union. The death of the Queen, the trigger that created a liminal space of uncertainty, is also one where her final ‘act’ of dying in Scotland is interpreted as potentially defining the path out of uncertainty and into a unified future.

In the examples presented above, the Queen is yet again strategically positioned as embodying Britishness, an argument used to reassert a particular notion of the national identity through emphasizing ‘what’ she embodied: core values (unity), group boundaries (Union) and standing (pride). The loss of the Queen, as a key positive representation of the monarchy amid recent negative events surrounding the royal family (including the departure of Harry and Meghan from the royal family, the scandals involving Prince Andrew and the popular tales of Charles and Camilla narrated through television programmes such as ‘The Crown’) makes her passing a key rupture. As one participant in London remarked ‘I think Charles is a good guy, but he'll never have the gravitas that the Queen had, you know, either in this country or globally, you know, I think the influence and the respect that she had… [I: yeah], is unique and won't ever be repeated’.

For those participants who equated the Queen with the monarchy, the transformative impact of the liminal occasion is limited. Indeed, instead the moment becomes one of reaffirming the unifying role played by the monarchy and its relevance for the collective. The transformative ‘moment’ here is narrow in terms of the monarchy's role in British identity, and wide in terms of imagining a possible future of unity, with the monarchy at the core, as the ‘glue’. For those who see the Queen as unique however, the liminal occasion brings more of a paradox and possibility. It raises worries about the future with Charles who lacks the unique history and qualities of the Queen, and so opens up a space of tension – what kind of national character will he embody? The transformative potential of the liminal occasion is therefore given meaning through the rhetorical framing of the Queen vis‐a‐vis the monarchy; her uniqueness as a leader makes the potential impact on the national character more fundamental and the transition from between ‘worlds’ potentially more disruptive.

## DISCUSSION

Through analysing interview transcripts with those who participated in the national days of mourning in Scotland and England following the passing of Queen Elizabeth II, we explored the ways in which her death was experienced as a liminal occasion where participants strategically defined British national identity and its future. We see how participants position the Queen as representative of Britishness, her loss as a loss of their own national identity. Something once taken for granted is now put in question and needs to be (re)considered explicitly. We then see, in their use of temporal language and their emphasis on juxtaposing change and continuity with rupture and loss, how participants navigate through the liminal occasion between the reigns of Elizabeth and Charles as a collective. Their spontaneous and individual experiences are transformed by being part of a common ritualized pilgrimage which shapes, and thereby reduces, the possibilities afforded by the transition from past to future, ‘here’ to ‘there’.

The value of monarchy is asserted through its seemingly apolitical but crucially unifying position in the British national landscape, overcoming the venality of the political, bridging political party divides and national divides between England and Scotland. As liminality is ‘a world within and between worlds’ (Stenner, 2021) it is worth considering what ‘world’ is opened up, and negotiated, by the crowd. Set against a fractured socio‐political landscape embodied by Brexit and its unresolved tensions and challenges for UK democracy, the liminal occasion triggered by the sudden death of the Queen posed not only a threat to the role of the monarchy for the British national character, but importantly, it had the dangerous potential of becoming another event that further divided and weakened the British national identity, both within the group, and on the global scale (i.e. I think the influence and the respect that she had… [I: yeah], is unique and won't ever be repeated). Transitioning between worlds, from Queen to King, is accompanied by a desire to transform worlds in the process, where the loss of the Queen as a positive symbol of the national character is claimed and mobilized by the mourners as they imagine a future defined by unity and pride, rather than division and apology. Her death is as much met with sorrow, as it is with hope.

The emphasis on the monarch as the de facto unifying feature in an otherwise divided society makes the implicit assumption that support for the monarch in the United Kingdom is universal and uncontested. It also positions potential threats to the monarch as threats to British society. This constructs the Queen as a prototypical leader of the nation *as a whole*, rather than of a sectional interest within the nation. The liminal occasion created by the Queen's death and the loss of a popular royal figure created a moment where participants felt a need to reassert, rather than resist, the future role of the monarch as an essential part of British identity, society and state, symbolized through the metaphor of a ‘piece of glue’. Her death therefore raises a need, and even responsibility, among participants in the event, to carry this particular version of British identity forward in the face of potentially destabilizing change. In this way, the liminal occasion created by her passing is navigated with reference to the past, her embodiment of it and the public's role in bringing it into the future.

Talking about the Queen as apolitical, and as a cultural figure of positive nationhood enabled participants to frame their sense‐making as apolitical, particularly in reference to core values. This opens a space for national pride to be expressed and normalized as it is framed as a pride in a country's culture, history and values, rather than a pride that is associated with exclusive or narcissistic nationalism rooted in the subordination of others. We see that emphasis on traditional values, through respect for the monarchy, respect for authority, and stronger national pride are asserted as values which the Queen embodied, and which the public now must ‘pick up’ in her absence. In doing so, participants justify the need to reassert traditionalist values with reference to monarchy, history, and heritage (Billig, 2002).

Throughout the data, what ‘hums’ in the backdrop of the discussion (see Wohl et al., 2022) is the position that the monarchy should maintain its place in the future of the British nation and that this can potentially counteract what is a highly fractured country in the present. If, as we have argued above, the monarchy is constituted as Britishness and the loss of the monarch is a loss of Britishness (in ourselves as well as in the world) you cannot have a future Britain without the preservation of the institution of monarchy. This is neither contested nor debated. Rather, within the liminal occasion constructed to enable navigating through loss, it was an often‐unspoken premise. Through references to history, heritage and tradition, participants strategically navigated uncertainty and threats to collective continuity by emphasizing the need for the institutions behind the Queen to be part of the future. As such, the ‘not yet’ of the future was constructed with reference to the ‘no longer’ of the Queen's monarchy as necessarily containing a future king or queen.

In considering how socio‐political changes, such as the sudden death of a monarch, become liminal occasions where taken‐for‐granted notions of nationhood come to the surface, the concept of liminality offers a useful complementarity to existing social psychological theorizing of social identity change. Namely, it illustrates how transitional moments enable and become sites for collective coping with change and identity threat, where sense‐making around transition and loss is achieved with references to the past and the broader socio‐political landscape. Liminal occasions become moments where nostalgia thrives (Davis, 1979) and directs what the future should look like (Wohl et al., 2022). Yet, this strategic articulation of what the British character of the future should look like is subtle, defined by discussing the difference between the past and the present (Billig, 2002). In the language of liminal theory, the in‐between space of a transitional moment is selectively shaped by how the ‘no longer’ is given meaning and drawn into shape by what the ‘not yet’ will become.

Although the death of Queen Elizabeth II. represents somewhat of unique event in the context of contemporary Britain, its effects arguably extended across other parts of the globe (e.g. Twomey, 2022). Moreover, like the reactions brought about by the Queen's death, the passing of other significant national figures has been known to cause periods of national mourning and rupture to collective identities that play out on a global stage. For example, the passing of Nelson Mandela in 2013, who was viewed to have both unified the South African people and transformed their country, was commemorated on a national stage through rituals, and globally broadcast through media, reaffirming his significance for the national identity (Uimonen, 2015). Indeed, classic accounts of processes of liminality and transition have argued for the significance of political leaders as symbols linked with periods of transition and transformation (see Szakolczai, 2009) suggesting that the processes observed within our study have the potential to extend to other global contexts, events and experiences.

### Limitations and future research

The present study was a reaction to an unpredictable event, and we managed to successfully conduct research as events unfolded (Hoerst et al., 2022). Within half a week of the Queen's death, we managed to assemble a research team, design, and plan the study, obtain rapid ethical approval and begin gathering data. To do so, we drew on recommendations from responsive research on emergencies and crowds (e.g. Vindrola‐Padros et al., 2020), such as the importance of internal ongoing communications with the team (Recommendation 1), and flexibly (re)drafting the interview schedule to meet the contextual demands of the rapidly changing nature of the phenomenon under investigation (Recommendation 2). This was essential to conduct time‐sensitive but high‐quality ethnographic research. However, we also acknowledge that our research was enabled by the existence of a rapid‐ethics structure at Keele University.

Our philosophy was not to let the perfect be the enemy of the good and to grab what we could from those who attended the mourning events in Edinburgh and London. That we managed to do anything is little short of miraculous (and raising the question of how we can better plan and fund research on spontaneous events in the future). But we do not deny that what we have is limited: our interviews were necessarily short (people wanted to attend to the events, not speak to researchers) and we weren't able to collect personal data about our respondents. Nonetheless, what we did obtain was rich and clearly of deep meaning to those in attendance.

We acknowledge the absence of dissenting voices. We looked at those who, by making the effort of attending the mourning events, indicated their support (or, at least, non‐opposition) to the monarchy and state, and who consequently might have experienced this as more of a liminal occasion than those for whom Britishness is not defined with reference to the monarchy. This does not mean that others, in other places, did not see the moment as potentially transformative, who in the loss of Queen Elizabeth and before King Charles was enthroned, envisioned a future without the monarchy. What is interesting is how few people did express this position in public and – for the few who did – such expressions were seen and treated as illegitimate (as a generically uncouth act of ‘speaking ill of the dead’ and of further upsetting those in grief rather than a legitimate political stance). The question of what could and couldn't be spoken in this period, and of how a pro‐royalty position was construed as hegemonic, are two of several issues we are addressing in our wider project on crowds following the death of the late Queen Elizabeth II. and including data collected as part of the Coronation that followed in 2023. In this paper, however, we concentrated on showing how liminality is a useful concept for examining historical transitions and people's national identifications.

## CONCLUSION

Liminality, as a concept that captures the uncertainty and ambiguity of in‐between spaces, transitory moments of passages of life, enables psychologists to examine the psychological sense‐making that occurs to restore certainty, create continuity and project agendas onto the future. Utilizing a social psychological lens, liminality as a concept also allows us to explore not only personal but *social* transition, which is both experienced but also constructed through shared rituals. Through a rapid response ethnography, we were able to capture the raw moments within which a collective comes together, as part of a national ritual, to make sense of where ‘we’ go from here. Liminality offers a useful conceptual tool for addressing how temporality and change are negotiated in relation to a shared identity and how this sense‐making process is a project which brings with it political implications for the future.

## AUTHOR CONTRIBUTIONS

**Sandra Obradović:** Writing – original draft; conceptualization; formal analysis. **Nuria Martinez:** Writing – review and editing; writing – original draft; formal analysis; investigation. **Nandita Dhanda:** Investigation; formal analysis; writing – review and editing. **Sidney Bode:** Investigation; formal analysis; writing – review and editing. **Evangelos Ntontis:** Conceptualization; formal analysis; investigation; methodology; writing – review and editing. **Mhairi Bowe:** Investigation; writing – review and editing; formal analysis. **Stephen Reicher:** Conceptualization; writing – review and editing. **Klara Jurstakova:** Investigation; writing – review and editing; methodology. **Jazmin Kane:** Investigation. **Sara Vestergren:** Conceptualization; data curation; methodology; project administration; writing – review and editing.

## CONFLICT OF INTEREST STATEMENT

All authors declare no conflict of interest.

## Supporting information


Appendix S1

## Data Availability

The data that support the findings of this study are available on request from the corresponding author. The data are not publicly available due to privacy or ethical restrictions.
