# Supplementary material for: Mourning and orienting to the future in a liminal occasion: (Re)defining British national identity after Queen Elizabeth II's death
Source: Br J Soc Psychol. 2024 Oct 8;64(1):e12807. doi: 10.1111/bjso.12807 (PMC11600473; doi:10.1111/bjso.12807)
Supplement: Supplementary file 1 — Appendix S1 [file BJSO-64-0-s001.docx]

## **Supplemental materials for** Mourning and orienting to the future in a liminal occasion: (re)defining British national identity after Queen Elizabeth II’s death.

## S1. Interview script for approaching and engaging participants.

1. Participants will be approach by a member of the research team, the researcher will introduce themselves as a researcher and state that we are conducting a study on experiences of the crowd in relation to the Queens death. If the potential participant indicates interest, the researcher will inform them that: a) their participation will be anonymous, data will be stored at Keele University only accessed by the research team and treated with confidentiality, b) they can withdraw at any point during the interview, but not at a later stage due to anonymity (lack of recorded identifiable details), and c) ask for consent to record the short interview.
2. Recording will start, the researcher will ask for audio recorded consent to participate in the interview as well as use the comments/quotes from the interview before moving on to the questions.
   1. Theme: why do people assemble? Question: why have you come along today?
   2. Theme: who are the members of the crowd? Question: ‘how would you describe the people who are in the crowd – and how would you describe yourself.
   3. Theme: what are relations amongst crowd members? Question: what are things like between people who are here?
   4. Theme: have people come alone or with others? Question: are you here with others (and, if yes) does it bring you closer to them?
   5. Theme: What is the experience of the crowd? Question: how would you describe your feelings about being here?
   6. Theme: What is the impact of participation? Question: do you think these events tell us anything about who we are as a society?
   7. Theme: Do people relate the Queen’s mourning to their own personal experiences of mourning? Does being here make you think about people you have lost in your own life?
3. When the short interview is about to close the researcher will ask the participant if they consent to sharing their contact details for future studies (post-event longer interviews on experiences and understanding of the crowd during the Queen's mourning - ethics will be sought separately).
4. The participant will be thanked and offered an opportunity to ask or answer questions that have not already been addressed.

## S2. Additional information on interview participants.

Table X1. Interview ID, length, and number of participants.

| Interview ID | Length (minutes) | No participants |
| --- | --- | --- |
| IE01_220912 | 00:59 | 1 |
| IE02_220912 | 11:44 | 1 |
| IE03_220912 | 02:18 | 1 |
| IE04a_220912 | 11:18 | 3 |
| IE04b_220912 | 03:00 | Same participants as above (longitudinal) |
| IE05_220912 | 08:33 | 3 |
| IE06_220912 | 09:10 | 2 |
| IE07_220912 | 04:45 | 1 |
| IE08_220912 | 10:46 | 2 |
| IE09_220912 | 04:49 | 2 |
| IE10_220912 | 09:59 | 3 |
| IE11_220912 | 07:37 | 1 |
| IE12_220912 | 11:20 | 2 |
| IE13_220912 | 11:55 | 2 |
| IE14_220912 | 03:14 | 1 |
| IE15a_220912 | 01:44 | 1 |
| IE15b_220912 | 01:47 | Same participant as above (longitudinal) |
| IE16_220912 | 05:31 | 1 |
| IE17_220912 | 05:18 | 2 |
| IE18_220912 | 04:18 | 1 |
| IE19_220912 | 05:33 | 2 |
| IL20_220913 | 02:19 | 1 |
| IL21_220913 | 01:32 | 1 |
| IL22_220913 | 01:45 | 1 |
| IL23_220913 | 03:52 | 1 |
| IL24_220914 | 06:40 | 2 |
| IL25_220914 | 09:25 | 2 |
| IL26_220914 | 04:17 | 1 |
| IL27_220914 | 05:51 | 3 |
| IL28_220914 | 03:53 | 3 |
| IL29_220914 | 01:48 | 1 |
| IL30_220914 | 03:13 | 1 |
| IL31_220914 | 02:07 | 1 |
| IL32_220914 | 01:56 | 1 |
| IL33_220914 | 01:57 | 2 |
| IL34_220914 | 03:57 | 1 |
| IL35_220915 | 07:25 | 2 |
| IL36_220915 | 04:14 | 2 |
| IL37_220915 | 07:11 | 2 |
| IL38_220915 | 06:00 | 1 |
| IL39_220915 | 04:26 | 1 |
| IL40_220915 | 04:57 | 1 |
| IL41_220915 | 03:49 | 2 |
| IL42_220915 | 06:01 | 4 |
| IL43_220915 | 21:20 | 2 |
| IL44_220915 | 06:33 | 2 |
| IL45_220915 | 05:19 | 3 |
| IL46_220915 | 03:45 | 2 |
| IL47_220915 | 05:48 | 2 |
| IL48_220915 | 08:21 | 4 |
| IL49_220917 | 07:24 | 3 |
| IL50_220917 | 06:08 | 1 |
| IL51_220917 | 05:38 | 2 |
| IL52_220917 | 07:52 | 2 |
| IL53_220917 | 12:50 | 2 |
| IL54_220917 | 07:06 | 2 |
| IL55_220917 | 04:45 | 3 |
| IL56_220918 | 04:01 | 3 |
| IL57a_220918 | 15:35 | 3 |
| IL57b_220918 | 03:06 | Same participants as above (longitudinal) |
| IL57c_220918 | 08:15 | Same participants as above (longitudinal) |
| IL58_220918 | 01:49 | 2 |
| IL59_220919 | 24:.04 | 1 |
| IL60_220919 | 05:55 | 5 |
| IL61_220919 | 05:26 | 3 |
| IL62_220919 | 04:58 | 1 |
| IL63_220919 | 13:09 | 2 |
| IL64_220919 | 06:14 | 3 |
|  |  |  |
| **Total number of interviews:** 64   (66 if the longitudinal 3 counts as 3)  *Longitudinal interviews highlighted in red. | **Total dataset length**: 429 minutes, 34 seconds  (7h 9min 34sec) | **Total number of participants**: 122  **Single interviews**: 25  **Group interviews**: 39 |
